# Multi-expert ensemble ECG diagnostic algorithm using mutually exclusive–symbiotic correlation between 254 hierarchical multiple labels

**DOI:** 10.1038/s44325-024-00010-0

**Published:** 2024-07-02

**Authors:** Jiewei Lai, Yue Zhang, Chenyu Zhao, Jinliang Wang, Yong Yan, Mingyang Chen, Lei Ji, Jun Guo, Baoshi Han, Yajun Shi, Jinxia Zhang, Yundai Chen, Qianjin Feng, Wei Yang

**Affiliations:** 1 School of Biomedical Engineering, Southern Medical University, Guangzhou, China; 2 Guangdong Provincial Key Laboratory of Medical Image Processing, Guangzhou, China; 3 CardioCloud Medical Technology (Beijing) Co., Ltd., Beijing, China; 4 IT Department, Chinese PLA General Hospital, Beijing, China; 5 Department of Cardiology, Chinese PLA General Hospital, Beijing, China; 6 Department of Cardiology, General Hospital of Southern Theatre Command of PLA, Guangzhou, China

**Keywords:** Cardiology, Health care

## Abstract

Electrocardiograms (ECGs) are a cheap and convenient means of assessing heart health and provide an important basis for diagnosis and treatment by cardiologists. However, existing intelligent ECG diagnostic approaches can only detect up to several tens of ECG terms, which barely cover the most common arrhythmias. Thus, further diagnosis is required by cardiologists in clinical settings. This paper describes the development of a multi-expert ensemble learning model that can recognize 254 ECG terms. Based on data from 191,804 wearable 12-lead ECGs, mutually exclusive–symbiotic correlations between hierarchical multiple labels are applied at the loss level to improve the diagnostic performance of the model and make its predictions more reasonable while alleviating the difficulty of class imbalance. The model achieves an average area under the receiver operating characteristics curve of 0.973 and 0.956 on offline and online test sets, respectively. We select 130 terms from the 254 available for clinical settings by considering the classification performance and clinical significance, providing real-time and comprehensive ancillary support for the public.

## Introduction

**Fig. 1:**
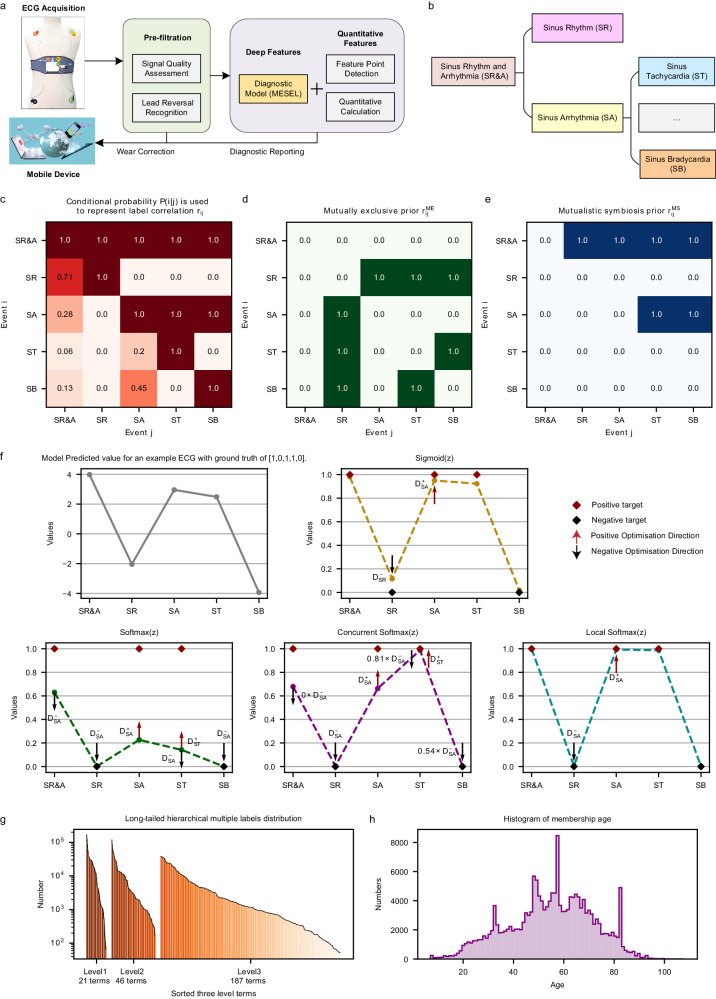
ECG diagnostic framework, dataset information, and mutually exclusive–symbiotic correlation of hierarchical multiple labels. **a** One-stop intelligent diagnostic framework for 12-lead wearable ECG. **b** Example of hierarchical labels in our ECG database. **c** Conditional probability matrix representing the concurrence of multiple labels. **d** Mutually exclusive correlation matrix of multiple labels computed from the conditional probability matrix. **e** Mutually symbiotic correlation matrix of hierarchical labels computed from the conditional probability matrix. **f** Motivation and schematic diagram for local softmax. The subfigure shows a prediction vector for an example ECG, and the results of activating this vector using four different activation functions: sigmoid, softmax, concurrent softmax, and local softmax. **g** Three hierarchies of labels, all of which are consistent with the long-tailed distribution. **h** Histogram of age distribution of 87,973 members.

Electrocardiograms (ECGs) are electrophysiological signals produced by a potential difference, as measured by electrodes placed on the surface of the human skin. They are the most common, convenient, and inexpensive non-invasive tests of the cardiovascular system. Cardiologists leverage ECGs to diagnose various abnormal cardiac activity states by analyzing the corresponding waveform changes, with timely intervention known to improve the health of patients and the survival prognosis. Wearable ECG devices, which have user-friendly designs and compact sizes, can be worn by the subject 24/7, and the collected recordings can be automatically uploaded to a server^1^. Compared with hospital-based ECG machines, which collect ECGs over just a few minutes, wearable devices are more likely to collect non-rhythmic or transient data, such as premature ventricular beats, premature atrial beats, and other precious arrhythmia signals, in a few days of collection time. Typically, the non-rhythmic or transient class is also the rare class. For deep learning approaches, collecting more data on rare classes is a fundamental way to improve the classification performance of models for rare classes. At the same time, the prolonged use means that wearable ECG devices can collect abundant data, aligning with the advantages and requirements of deep learning methods for mining information to produce intelligent diagnostic models that enable clinical decision-making. Cardiovascular disease has far surpassed cancer as the leading cause of death worldwide^2^, and with increasing life expectancy, the aging of the population is gradually becoming a major problem in the field of healthcare across the world. To reduce the workload of cardiologists and realize intelligent monitoring and telemedicine, the need for clinically applicable ECG diagnostic approaches such as the one shown in Fig. 1a is increasingly urgent in modern society.

Before the era of deep learning, the scarcity of digital-format ECGs meant that many studies analyzed and processed ECGs at the heartbeat level based on the physiological waveform features^3,4^ and statistical features^5,6^, using small-scale datasets such as the MIT-BIH Arrhythmia Database^7^ and PTB Diagnostic ECG Database^8^. The classification performance of such approaches is limited by the richness of manually designed features, and small dataset sizes lead to poor reliability and generalization performance, making these models difficult to use for clinical assessment^9^. With developments in technology and the progress of society, the volume of ECG data has exploded^10–13^, and the research object of associated studies has gradually switched from the heartbeat level to the segment level, lasting several seconds or even minutes^14^. In addition to classical convolutional networks^15^, some studies have used feature extractors such as recurrent networks^16^ and Transformers^17^ to combine local features with global temporal features to process ECG data. The convolutional network model developed by Hannun et al.^18^ can detect and recognize 12 rhythm classes in single-lead ECGs and has achieved diagnostic performance at the level of cardiologists. Natarajan et al.^19^ proposed a wide and deep Transformer that combines manually designed features with automatically learned features from deep learning, enabling the diagnosis of 27 rhythm classes from 12-lead ECGs. Our previous work used a transfer learning model to introduce implicit knowledge of the ECG itself, enabling real-time diagnosis of 60 ECG diagnostic terms from 12-lead wearable ECG data^20^. However, the number of ECG terms that can be recognized by the abovementioned approaches is relatively small, and correlation between multiple labels^21^ is not used to improve the classification performance, which requires further diagnosis and annotation by cardiologists in a clinical setting, making it difficult to actually meet their diagnostic needs. Our task requires the recognition of up to 254 labels, so it is necessary to explore how to effectively exploit the label correlation of hierarchical multiple labels.

Computer-aided diagnostics are essentially a classification task. When an ECG has only one label, the task is multi-classed and there is no correlation between labels, and the loss is generally softmax, which uses very strong prior knowledge that each class is mutually exclusive to the remaining classes. When an ECG has more than one label, the task is multi-labeled^22^, and the loss is usually sigmoid, which is an S-shaped monotonically increasing function that maps the output values of the model to the range 0–1, with no use of multi-label correlation. For multi-label classification tasks, there are two main ways to exploit the correlation. The first is via specially designed network architectures, such as hierarchical multi-label classification networks^23^, tree LSTM^24^, graph neural networks^25^, and label decoupling networks^26,27^. However, this way of improving the architecture of neural networks to use correlation is implicit, which means that the interactions between the information for each class occur within the network architecture, and this process is difficult to examine and intervene. The second way to take advantage of label correlation is to use it at the loss level in a direct style, such as the concurrent softmax^28^ and seesaw loss^29^ functions, which are improved versions of softmax, or through the ranking loss, such as with LESP^30^ and multi-label softmax^31^. However, these efforts mainly address data imbalances and target improved classification performance. As a result, they are unable to solve the following two issues (see Fig. 1b): (1) Two terms that are mutually exclusive, such as sinus tachycardia (ST) and sinus bradycardia (SB), should not be diagnosed from a single ECG at the same time; (2) The diagnostic result from an intelligent ECG model should include a complete chain of hierarchical labels. When diagnosing a third-level label like ST, the model should also diagnose its second-level label sinus arrhythmia (SA), and its first-level label sinus rhythm and arrhythmia (SR&A), based on the ECG. Similarly, when the first-level label SR&A is diagnosed, at the same time, model should either give a diagnosis of second-level label sinus rhythm (SR) or a diagnosis of third-level label ST or SB. The most straightforward way to address these two issues is to use correlation matrices (see Fig. 1c–e) at the loss level to constrain the prediction outcomes of the diagnostic model.

For the former issue, our solution strategy is to improve softmax to make it suitable for multi-label classification task. The vanilla softmax is often used in multi-class classification tasks, taking the value of the positive class to suppress the values of all remaining classes, as shown in Fig. 1f. However, for multi-label classification tasks, where there is more than one positive label for the same sample, optimization for one positive class strongly conflicts with the suppression from other positive classes to it, making softmax far inferior to the sigmoid function. Concurrent softmax^28^ mitigates the suppression of one positive class to the other positive classes that co-occurrence with it according to the concurrent correlation matrix, as illustrated in Fig. 1c, constructing a bridge between softmax and the multi-label object detection task. However, this suppression is only mitigated, and does not disappear. Hence, there are still conflicts in the direction of gradient optimization in the positive class. Although concurrent softmax is better than softmax, it is still not as good as sigmoid for our multi-label classification task. Therefore, we propose local softmax based on mutual exclusion correlation, as illustrated Fig. 1d. In the case of considering only the five classes shown in Fig. 1b: with local softmax, the output value of the positive class SA only suppresses its mutual exclusion classes SR, and no longer suppresses its co-occurrence classes SR&A, ST and SB, restricting the suppression range of softmax from global to local, and making it suitable for multi-label classification tasks.

In addition to the mutually exclusive relationship between multiple labels, there is a mutually symbiotic relationship among hierarchical labels. In the ECG database with three levels shown in Fig. 1b, all four child nodes under which the parent node SR&A, is its symbiotic classes. Thus, for the latter issue, we propose the symbiotic ranking regularizer (SRR) based on the mutually symbiotic correlation shown in Fig. 1e, using the form of the pairwise ranking loss: the output of the model at the parent node must be greater than the output value at any of its child nodes. There are two cases here: when the parent node is 1, there must be at least one child node that gives a value of 1; when the parent node is 0, all of its child nodes must be 0. Analogously, the SRR is like a fruit tree, where the parent node is a branch, and the child nodes are its fruits. If there are many fruits, the branch must be strong and sturdy; if the branch is dry, there will be no fruit. The SRR is based on the mutually symbiotic correlation matrix, which is statistically pre-counted and is not used as the ground truth of ECG recordings during training, and is therefore unable to reduce the training error of the model. The SRR directly constrains the output values of the model at the loss level in the expectation that the model will output more reasonable predictions, thus reducing the generalization error.

As we have up to 254 ECG terms as classification labels, the disparity in the size of positive samples from different classes will be large, leading to the data imbalance problem illustrated in Fig. 1g. There are several ways to deal with unbalanced data^32^: cost-sensitive learning, which sets class weights according to the proportion of positive samples per class^33^; under- or over-sampling, which adjusts the data distribution during training^34^; focal loss, which focuses the model on harder-to-learn samples^35^; balanced^36,37^ and equalization loss^38^, which simulate balanced distributions via logit adjustment; transfer learning, which introduces external information and knowledge to downstream task^39^; and ensemble learning, which aggregates the strengths of multiple experts^40^. In this study, the local softmax function uses the mutually exclusive relationship between multiple labels, SRR uses the mutually symbiotic relationship between hierarchical labels, and the sigmoid function does not use any label relationship. These three losses complement each other and are in line with the “good but different” essence of ensemble learning. The aim of ensemble learning is to reduce the overall variance and obtain better classification performance by training multiple weak base learners, which are as different as possible, before finally aggregating them. The SADE^41^ ensemble learning approach was trained with the three different losses to simulate three corresponding distributions, which are long-tailed, balanced, and inverse, respectively, and could adapt to various test sets with unknown real-world distributions to deal with the data imbalance problem. We improve SADE use local softmax and SRR, and further propose the mutually exclusive–symbiotic ensemble learning (MESEL) method, which combines the advantages of diverse experts with better classification performance.

Here, we improve the intelligent diagnostic model of the one-stop wearable 12-lead ECG diagnostic framework^20^ as shown in Fig. 1a: (i) home users upload their acquired ECG data via the wearable device to a server; (ii) the signal quality assessment model determines whether the signal quality is acceptable or not^42^, and the lead reversal model determines whether the device is being worn properly^20^, so as to correct the wearing condition of users and pre-filter the ECGs to be diagnosed; (iii) the MESEL diagnostic model proposed in this paper is used to predict the associated ECG terms; (iv) the feature point detection model is applied to detect the start and end points of the physiological waveform of the recording^43^, and then the quantitative indicators of traditional ECG diagnostics are calculated; (v) this two information are merged to generate a diagnostic report that is returned to the uploader. The diagnostic model developed in this study uses the MESEL architecture with a total of 12 losses: sigmoid, symbiotic sigmoid, local softmax, and symbiotic local softmax as the four basis losses, each of which produces three logit adjusted methods for long-tail, balanced, and inverse distributions. These 12 losses are used to construct a multi-expert aggregated model suitable for the hierarchical multi-label classification task, capable of performing real-time intelligent diagnostics on 245 ECG terms. The classification performance of the model is evaluated on a reserved offline test set, and a prospective test is conducted to evaluate the generalization performance of the model using an online test set. By considering both the intelligent diagnostic performance and clinical use significance for each term, we select a subset of 130 terms and deploy the model to provide high-quality services for the public.

## Results

### Dataset

We constructed a large-scale 12-lead wearable ECG dataset consisting of 191,804 recordings collected from 2016–2023 using the same type of wearable ECG device (belonging to the Mason-Likar system). These recordings were manually screened and trimmed to ensure that the waveform information was useable, i.e., having a duration of 15 s, a digital sampling frequency of 500 Hz, and a total of 12 leads: I, II, III, AVR, AVL, AVF, and V1–V6. These data were collected from three sources: by patients examined in a hospital using the wearable device; by users in their home environment wearing the same device; and by volunteer diagnostic activities with the company and the hospital. All data were collected within mainland China from a total of 87,973 people (62.91% male, 37.09% female; aged from 7–108 years old with a mean of 54.69 years and a median of 55 years; see Fig. 1h).

The dataset is multi-labeled and hierarchical, with three levels containing 21, 58, and 288 labels, respectively; the affiliations are presented in Supplementary Table 1. After removing duplicate terms, we used MESEL to recognize 254 labels for which the positive sample size exceeded 50, with 21, 46, and 187 labels in each level, respectively, as shown in Fig. 1g. The gold standard annotation of all ECGs was produced by a committee of cardiologists: for each ECG, two cardiologists diagnosed the data, and three different senior cardiologists reviewed the data. If the result was in agreement, the annotation was confirmed; if there was some disagreement, an arbitration group discussed the data and made a final decision and annotation.

There are three main types of interference faced by wearable ECGs: baseline wander, power frequency interference, and myoelectric interference. Baseline wander is generally below 0.5 Hz and is easily removed using digital filters. Due to the specialized electromagnetic isolation design of the wearable ECG device, the power frequency interference at 50 or 60 Hz is virtually non-existent. Myoelectric interference is generally a high-frequency signal with frequencies above 35 Hz. Abnormalities in ECG waveforms are typically also high-frequency signals, making it difficult to distinguish between the two types of information using only digital filters. We, therefore, filtered all ECG recordings using a high-pass filter with a low cutoff frequency of 5 Hz to remove baseline drift and preserve high-frequency diagnostic information. Therefore, a 5th-order Butterworth high-pass filter with a lower cutoff frequency of 0.5 Hz is used as a pretreatment for all ECGs. In addition, the quality of the ECG may be bad or contain no diagnostic information in cases of poor lead electrode contact or the incorrect wearing of the device. We developed a data quality assessment model^42^ to evaluate the quality of the ECGs to be diagnosed and pre-filter ECGs of substandard quality. The lead electrodes of the wearable device need to be positioned by the users themselves, so there is some possibility of lead reversal. The lead reversal recognition model^20^ was developed to recognize the vast majority of lead reversal cases. These two models are deployed to the mobile device, warning about and correcting the incorrect wearing of the device in a timely manner at the data collection stage.

Of the 191,804 recordings obtained, we randomly selected 182,324 ECGs as the training set and the remaining 9480 ECGs as the offline test set for evaluating the classification performance of the model in a laboratory setting. The dataset splitting criterion was based on the identity document (ID) of the members, thus guaranteeing that the member IDs in the training set could not appear in the offline test set to avoid information leakage. We deployed the model on a server to provide online real-time intelligent diagnostic services on 13,281 ECGs uploaded by users during from August to October 2023. This served as an online test set for assessing the reliability of the model for application in a clinical setting.

### Metrics

We evaluated the classification performance of the model using six indicators: area under the receiver operating characteristic curve (AUROC), area under the precision–recall curve (AUPRC), sensitivity, specificity, F1-score, and accuracy. AUROC indicates the ability of the model to correctly classify all samples. AUPRC, also known as average precision (AP), is sensitive to the number of positive samples and is the main observation indicator for our experiments, indicating the ability of the model to correctly detect positive samples. The sensitivity, specificity, F1-score, and accuracy are calculated after using pre-selected thresholds that binarize the predicted values from the model outputs. Sensitivity, also known as recall, is the ratio of positive samples that can be detected by the model to all true positive samples, with higher values indicating that fewer samples have been missed. Specificity is the ratio of true negative samples that can be detected by the model to all negative samples, with higher values indicating that fewer negative samples are judged as false positives by the model. The F1-score comprehensively considers the recall and precision of a class. Similar to AUPRC, this metric is sensitive to the number of positive samples, and is the most used assessment indicator for classification tasks. Accuracy is the ratio of the number of samples correctly classified by the model to the total number of samples, and reflects the classification performance of the model holistically. Given that each ECG may have more than one label at the same time, we evaluated the model statistically in two ways: label-wise assessment for each class individually, and exam-wise assessment for each ECG individually. All indicators take values in the range 0–1, with larger values indicating better classification performance by the model.

### Experiment

We used the BCE as the loss, and sigmoid as activation function of the multi-scale convolutional network to establish a baseline, and analyzed the effectiveness of softmax, concurrent softmax, local softmax, and SRR on the classification performance of the model. We observed the distribution in micro AUPRC of the 12 “good but different” losses on the 254 labels, and use ablation experiments to analyze the improvement of classification performance in different combinations of them. To validate the diagnostic performance of MESEL, we show various evaluation metrics of the model on offline and online test sets, with both label-wise and exam-wise assessments. We use the proposed MESEL to improve six existing approaches that utilize hierarchical multi-label correlation, and analyze the effectiveness of our approach using correlation and the compatibility for other approaches. Finally, we reproduce MESEL on an open-source dataset PTB-XL, and its performance outperforms five previous state-of-the-art (SOTA) approaches.

### Contribution of mutual exclusive correlation

**Fig. 2:**
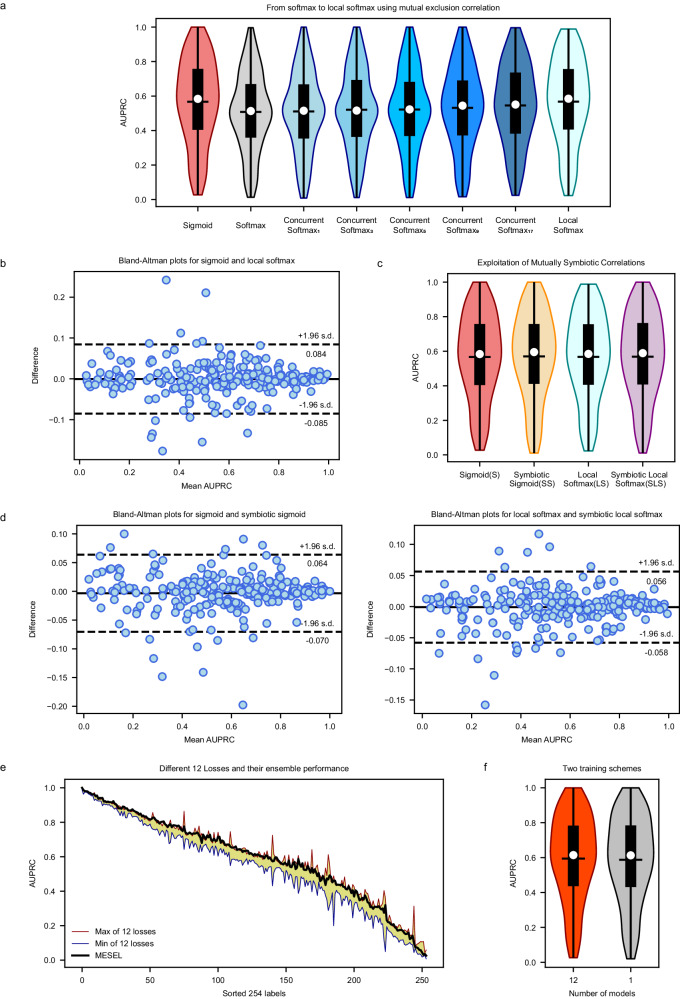
Classification performance and analysis of the proposed MESEL and its four basis losses. **a** AUPRC distribution of sigmoid, softmax, concurrent softmax, and local softmax. In our multi-label classification task, the performance of softmax is far inferior to that of sigmoid, and the performance of concurrent softmax gradually improves as the parameter ${\rm{\tau }}$ increases from 1 to 17. The classification performance of local softmax reaches the level of the baseline loss sigmoid in macro AUPRC. **b** Bland-Altman plots for sigmoid vs. local softmax, and both of them do not have an overwhelming advantage over each other in micro AUPRC across all labels, with differences even exceeding 20% in some classes. **c** AUPRC distribution of sigmoid (S), symbiotic sigmoid (SS), local softmax (LS), and symbiotic local softmax (SLS), and their classification performance is at an akin level with no significant statistical difference in macro AUPRC. **d** Two Bland-Altman plots, one for S vs. SS and the other for LS vs. SLS, and both of two plots show that the losses to be compared differ significantly in micro AUPRC across all labels. **e** AUPRC distribution of 254 labels from 12 “good but different” losses with yellow background, and the ensemble performance of MESEL in bold black line. **f** Comparison of private backbone and shared backbone schemes on AUPRC. The private backbone scheme requires 12 models to be trained, which is worse in terms of computational resources but results in better performance. The shared backbone has only one model, making it easy to train and requires less computational resources, but produces worse performance.

To solve the issue of “the model should not diagnose two mutually exclusive labels for one ECG”, we proposed local softmax, which takes advantage of the mutually exclusive correlation between multiple labels as Fig. 1d. We constructed the baseline with MSDNN as the encoder and sigmoid as the activation function, as shown in Fig. 2a, and its average AUPRC is 0.567. Also, in Fig. 2a, the average AUPRC of softmax is 0.508, decreases by 5.9% compared with the baseline, indicating that the treatment of all classes as mutually exclusive is indeed inappropriate for multi-label classification tasks. We use parameter ${\rm{\tau }}$ improves the concurrent softmax, and compared with the baseline, resulting in average AUPRCs are 0.511, 0.520, 0.522, 0.532, and 0.545, respectively, when ${\rm{\tau }}$ is set to 1, 3, 5, 9, and 17. Overall, the classification performance of concurrent softmax better than softmax, but is worse than sigmoid, which suggests that concurrent softmax alleviates the suppression of the current positive class on its co-occurrence positive classes based on the correlation of multiple labels, although there is still room for improvement using the parameter ${\rm{\tau }}$. As this parameter increases, the classification performance of the improved concurrent softmax gradually approaches that of the baseline. Letting ${\rm{\tau }}$ tend to infinity transforms the concurrent softmax into local softmax, where the current positive class only suppresses its mutually exclusive classes. Compared with softmax, this suppression is reduced from the global scale to the local scale based on mutually exclusive correlation. The average AUPRC of local softmax mirrors the baseline-level performance, achieving a value of 0.567.

Although the advantage of local softmax over sigmoid is not significant in view of average AUPRC, also named macro AUPRC, there is still difference should not be overlooked between sigmoid and local softmax from the micro view of AUPRC across each class. As shown in Fig. 2b, sigmoid and local softmax do not have an overwhelming advantage over each other, and the consistency of the classification performance on the 254 classes is poor, with the maximum difference even exceeding 20% in some classes. This is exactly what we want to see happen, which is consistent with the core idea of the “good but different” for ensemble learning, and it also is the key to why MESEL is able to improve classification performance.

### Contribution of mutual symbiotic correlation

To solve the issue that “the diagnosis of model for of one ECG should be a complete chain containing different hierarchical labels”, we proposed the SRR, which makes the prediction of model for the parent node larger than any of its children, which takes advantage of hierarchical label affiliation, also called mutually symbiosis correlation. The SRR can be applied to both sigmoid (S) and local softmax (LS) to give symbiotic sigmoid (SS) and symbiotic local softmax (SLS), respectively. As shown in Fig. 2c, the average AUPRC of these four methods is 0.567, 0.570, 0.567, and 0.568, respectively.

Similar to the findings in the above section, SS compared to S, and SLS compared to LS, do not have a significant improvement in macro AUPRC, but according to Fig. 2d, both two Bland-Altman plots show that the losses to be compared differ significantly in micro AUPRC across all labels, with the maximum difference even exceeding 10% on some classes. This suggests that both sigmoid and local softmax are altered to a greater extent when combined with SRR, which gives them the potential to be used in ensemble learning for improving classification performance.

### “Good but different” 12 losses

In the SADE ensemble learning architecture^41^, the original pipeline has three logit adjusted losses derived from softmax: long-tailed, balanced, and inverse for multi-class classification tasks. To accommodate multi-label classification tasks, we replace the base softmax loss with the sigmoid, local softmax, symbiotic sigmoid, and symbiotic local softmax, the latter two of which are obtained by adding SRR to the first two. To deal with the data imbalance, we also apply the three logit-adjusted methods of long-tailed, balanced, and inverse to these four base losses, resulting in a total of 12 losses. These can be regarded as 12 different experts working together to concentrate on the intelligent diagnosis task, and the average AUPRC values of these 12 losses are 0.567, 0.572, 0.575, 0.570, 0.569, 0.571, 0.567, 0.570, 0.572, 0.568, 0.568, and 0.571, respectively. As shown in Fig. 2e, to avoid too busy visual representation, we only drew the range of their distribution, indicated by the yellow-filled area. The maximum, mean, and minimum values of the differences between these 12 losses on various classes are 32.5%, 7.0%, and 0.5%, respectively. Although they are not significantly different in macro average AUPRC, each of these experts has a different focus on the terms to be diagnosed, suggesting that these models have great potential to improve the overall classification performance in the form of ensemble learning. After aggregating them using the averaging method, the final classification performance of MESEL of bold black line in Fig. 2e, essentially meets or exceeds the maximum performance of these 12 losses in micro AUPRC across most labels.

### Ablation analysis of classification performance by various aggregations

After obtaining these 12 expert models, the final result is obtained by simply averaging their respective predictions. We use ablation experiments to explore the contribution of 12 losses in different combinations, as shown in Table 1, where L, B, and I stand for three logit adjusted methods: long-tailed, balanced, and inverse, respectively. The baseline is S-L, achieved an average AUPRC of 0.567. The improvement is 1.2% when using the aggregation of S-L and SS-L, attributed to the proposed SRR; the improvement is 1.3% when using the aggregation of S-L and LS-L, attributed to the proposed local softmax, indicating that the exploitation of mutual exclusive–symbiotic correlation between hierarchical multiple labels improves the classification performance of the model. The average AUPRC improves by 2.0% over the baseline with the aggregation of the S-L, LS-L, SS-L, and SLS-L, indicating that the proposed local softmax and SRR are better when used in combination than individually. Finally, the best-performing aggregated model, which incorporates all 12 losses, achieves a 2.7% improvement over the baseline. This increase of 0.7% over the improvement of 2.0% given by the aggregation of four models comes from mitigating the data imbalance. Therefore, these 12 losses adhere to the core principle of “good but different” in ensemble learning. Aggregating them not only leverages the mutually exclusive–symbiotic correlation of hierarchical multiple labels but also addresses data imbalance, contributing to a practical enhancement of the ensemble model’s classification performance.

**Table 1:** Ablation experiments exploring the contribution of 12 losses in different combinations

Sigmoid (S)			Symbiotic sigmoid (SS)			Local softmax (LS)			Symbiotic local softmax (SLS)			AUPRC
L	B	I	L	B	I	L	B	I	L	B	I	
✓												0.567
✓			✓									0.579
✓						✓						0.580
✓			✓			✓			✓			0.587
✓	✓	✓	✓	✓	✓	✓	✓	✓	✓	✓	✓	0.594

### Comparison of two training schemes

**Fig. 3:**
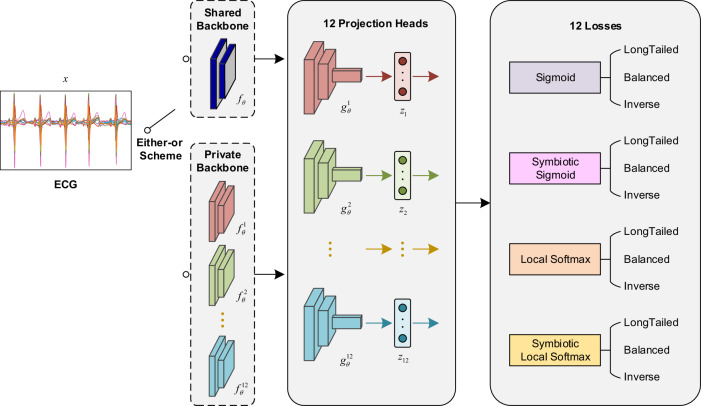
Architecture of MESEL. The input is an ECG recording with a duration of 15 s, and the output is 12 sets of prediction vectors of length 254. The backbone of the network has two available options: the shared backbone and the private backbone. The shared backbone requires only one model to be trained, which has 12 projection heads and outputs corresponding to the 12 losses, and the total loss is the sum of these 12 losses. The private backbone uses the 12 losses to train 12 models, respectively. The final prediction of the input ECG is the average of the 12 predictions.

As shown in Fig. 3, the backbone of the MESEL has two available schemes: the shared backbone and the private backbone, and the results of them can be seen in Fig. 2f, with average AUPRCs are 0.588 and 0.594, respectively. Training multiple networks separately with the 12 losses and then averaging the predictions outperform a single network trained with these losses at the same time. Therefore, in the pursuit of better performance, the first choice is to train multiple models; under computational, memory, or prediction time constraints, it is sufficient to train a single model using multiple loss functions at the same time. In each scheme in Fig. 3, the aggregation of multiple experts compared to a single-expert model in a non-ensemble way, will increase the number of parameters and computational load of the model, and reduce the diagnosis speed. Nevertheless, MESEL is a lightweight model, with only 2.07 M parameters, and the diagnosis time of MESEL for a single ECG averages 0.2 s, with a maximum of 0.3 s in continuous operation. This enables real-time diagnosis, meeting the demands of practical applications.

### Diagnostic performance

As listed in Table 2, the average AUROC, AUPRC, and F1-score of the model are 0.973, 0.594, and 0.585, respectively, for the 254 classes in the offline test set; the average sensitivity, specificity, F1-score, and accuracy on the online test set are 0.561, 0.978, 0.519, and 0.969, respectively. The specific values for each class are displayed in Supplementary Tables 2 and 3. The predicted values of the model output are mapped to 0–1 in the value domain after passing through the activation function, and then we manually fine-tune the threshold in the neighborhood of the break-even point. Ultimately, the output vectors are binarized by the threshold to obtain the intelligent diagnostic results^20^. The fine-tuned thresholds tend to increase the sensitivity of the ECG diagnosis, leading to a higher false positive rate. This is actually in line with the expectations of cardiologists: false positive samples will eventually be signed off by a human expert, which is only a slight increase in workload; missed ECGs will cost the patient a valuable early diagnosis and cannot be afforded. The F1 scores for the online test set dropped by 6.6% compared with the offline test set. This is mainly because there are more negative samples in the real-life scenario, resulting in more false positives close to or even greater than true positives. This result is acceptable based on the confusion matrix for each class.

**Table 2:** Diagnostic performance of the MESEL on the offline and online test sets

Test set	Samples	Eval-wise	AUROC	AUPRC	TP	TN	FP	FN	Sen	Spe	F1	Acc
Offline	9480	label	0.973	0.594	338.1	8906.0	146.0	89.9	0.618	0.982	0.585	0.975
		exam	-	-	9.1	239.6	3.9	2.4	0.785	0.984	0.727	0.975
Online	13282	label	0.956	0.509	431.2	12435.9	258.7	156.2	0.561	0.978	0.519	0.969
		exam	-	-	8.2	238.8	5.0	3.0	0.746	0.979	0.681	0.969

In the exam-wise assessment, there are an average of 8–9 positive labels and nearly 240 negative labels per ECG. The vast majority of the labels are correctly identified on both the offline and online test sets, with accuracy levels of 0.975 and 0.969, respectively. This suggests that our model is unlikely to annotate an ECG with many labels, which would cause unnecessary patient anxiety, and is still reliable when used in real-world cases.

### Clinically useful terms

The ECG database we constructed contains more than 300 diagnostic terms, and our aim was to recognize all of them via the machine learning model for clinical applications. However, limited by the number of positive samples, we selected 254 ECG terms for which the positive sample size was >50 in the database for this academic classification study. Hence, it is unlikely that all classes of indicators will meet expectations. In our experience, there are two factors that affect the intelligent classification performance for a class: the positive sample size and the inherent difficulty of the classification task itself. Generally, when there is insufficient data, the sample size is the main factor in dragging down the classification performance. Collecting more samples from rare classes can widen the knowledge of the model and increase the number of patterns that it knows how to solve. As the size of the dataset increases, the inherent difficulty of the classification task itself becomes the main factor limiting the performance of the model. Thus, we must improve the model with better feature representation, and fuse domain knowledge of ECG diagnostics within the model. In addition to diagnostic performance, clinical significance is a consideration of whether a particular term can be used in its clinical sense. As shown in Fig. 1b, once the third-level terms ST and SB are recognized by the model within an acceptable tolerance range, the importance of the corresponding second-level term CSA and first-level term SR&A rapidly declines. These higher-order terms could be masked out in practical applications, although they exist to aid the training of the model. Given these two factors, we selected a subset of 130 terms that have particular clinical relevance, which will be dynamically adjusted in future work based on feedback from cardiologists, and they are shown in red bold font in Supplementary Table 1.

### Improvement of existing approaches using MESEL

The proposed MESEL is an ensemble learning framework that uses the correlation among hierarchical multiple labels. The feature extractor of MESEL is a multi-scale convolutional neural network with 12 classifiers or experts, which is highly compatible with existing deep learning methods. As presented in Table 3, we compare baseline and other six representative approaches, which utilize label correlation in different ways, and improve them using MESEL. Sigmoid is the baseline for our multi-label classification task; HMCNF^23^, MLGCN^25^, Chang’s method^26^, and LDM^27^ are specially designed network architectures; LSEP^30^ and MLSoftmax^31^ are loss functions. HMCNF is designed to utilize the correlation of hierarchical labels, and outputs each hierarchical label layer-by-layer during the forward propagation of the data stream, fusing the global and local label information of various hierarchies. MLGCN focuses on the correlation between multiple labels to design a unique classifier for multi-label classification tasks. Chang’s method decouples the label correlations of different hierarchies in the classifier and reinforces the high-level label features with low-level label features. LDM decouples all labels and reduces the complexity of the hidden feature space. LSEP uses the pairwise ranking loss to reduce the example-wise ranking error. Finally, MLSoftmax reduces both the inter-label and intra-label ranking errors.

**Table 3:** Comparison of baseline and six approaches that utilizes label correlation before and after improvement using MESEL on our offline test set

Methods	Vanilla		Integration with MESEL (ours)	
	Paras (M)	AUPRC	Paras (M)	AUPRC
Sigmoid	2.07	0.567	12.78	0.588
HMCNF^23^	2.20	0.573	14.37	0.592
MLGCN^25^	2.26	0.559	15.07	0.578
Chang, 2021^26^	2.24	0.562	14.92	0.582
LDM^27^	13.17	0.574	145.99	0.591
LSEP^30^	2.07	0.591	12.78	0.603
MLSoftmax^31^	2.07	0.589	12.78	0.602

We reproduced these six approaches and tested them on the offline test set. In addition, we integrated them into the MESEL framework. As shown in Table 3, the improved ensemble learning networks produce significant improvements over the single networks in terms of the average AUPRC, with minimum, average, and maximum improvements of 1.2%, 1.7%, and 2.1%, respectively. This demonstrates that our method of explicitly exploiting the mutually exclusive–symbiotic correlation between multiple hierarchical labels at the loss level is effective.

### Open-source dataset evaluation

To evaluate the effectiveness and generalizability of the proposed MESEL on external data, we applied it to the open-source dataset PTB-XL^12^ (https://physionet.org/content/ptb-xl/1.0.3/). PTB-XL dataset comprises 21,799 clinical 12-lead ECG records of 10 seconds length from 18,869 patients, where 52% are male and 48% are female covering ages ranging from 0 to 95 years (median 62 and interquartile range of 22). This dataset is multi-labeled, with two common classification schemes: 5 superclasses and 23 subclasses. MESEL was originally developed to deal with correlations between hierarchical multi-labels, and is more effective when the label relationships are complex, so we adopted the classification scheme of 23 classes. The training and test sets were divided following the officially recommended mode, which includes 19,601 training samples and 2,198 test samples. Since the labels in PTB-XL do not have a hierarchical affiliation, the main losses used in reproducing MESEL are only sigmoid and local softmax. We compared with 5 existing arrhythmia classification approaches on the 23 subdiagnosis, and the AUROC and AUPRC were 0.936 and 0.563 as in Table 4, respectively, which outperformed the 5 previous approaches. The results show that MESEL can indeed handle the correlation between multiple labels reasonably, and improve the classification performance.

**Table 4:** Comparison of classification performance between previous SOTA methods and ours MESEL on the PTB-XL dataset in the sub-diagnosis class-level

Methods	Year	Sub classes (23)	
		AUROC	AUPRC
ATICNN^47^	2020	88.2	-
DMSFNet^48^	2020	89.7	-
XResNet 1D 101^49^	2020	92.9	-
ASTLNet^50^	2023	93.2	-
MSDNN^20^	2023	93.3	52.8
**MESEL**	**ours**	**93.6**	**56.3**

The bold line represents the classification performance of the proposed MESEL, outperforming these methods in both AUROC and AUPRC metrics.

## Discussion

We have proposed the local softmax function and the SRR. These functions explicitly use the mutual exclusion-symbiosis relationship between labels in the loss function, thus constraining the model predictions in the training stage and making them more reasonable. Furthermore, from the ensemble learning principle of “good but different,” these functions were aggregated in SADE architecture, which improved the classification performance while alleviating the problem of data imbalance. The model was tested in a clinical setting over a three-month period and showed no significant degradation in its generalization performance over the course of a complex application. We considered the classification performance and clinical significance of all 254 original terms, and selected the 130 ECG terms that are most clinically useful and can serve cardiologists and patients with a fine-grained, real-time, and accurate diagnostic performance. This addresses the growing demand and burden for cardiovascular health examinations in hospital check-ups as well as in home settings for continuous heart health monitoring. In summary, the multi-expert ensemble model provides a feasible solution for intelligent diagnosis of wearable ECGs, and acts as a reference for cardiologists and researchers in related fields. Our approach enables continuous monitoring and remote diagnosis of ECGs, thus improving the health of patients through early detection and timely intervention, reducing healthcare costs, and alleviating the healthcare burden on society.

In terms of the applicability, since all ECG recordings were collected within mainland China, and significant differences exist in ECGs collected from different demographics using different devices, hence our model is currently only suited for the general public in China, and does not have global applicability. To promote our model, we will collect more recordings from different lead systems and integrate existing open-source ECG datasets to build a larger heterogeneous dataset. We will also work on developing domain-adaptive algorithms to reduce the divide between different populations and devices, and enhance the adaptability of our model to more application scenarios.

The correlation matrix used in the experiments was statistically derived from the annotation of the established dataset, reflecting the co-occurrence probability of multiple labels in the form of conditional probabilities. This is a biased estimate of real-world events. Poor data collection for certain classes and missing or incorrect annotations mean that the correlation matrix may distort the estimation of multi-label relationships and conflict with the perceptions and experiences of human cardiologists. The correlation matrix can be manually modified by experienced cardiologists, but this task would become very tedious as the number of labels increases. Therefore, we did not make any changes to the correlation matrix. This is not problematic because our dataset is relatively large, and the pre-statistic correlation matrix is reliable enough, it is still fine to follow the existing process even for some small-probability events.

Algorithmically, local softmax requires every class to have at least one mutually exclusive class. When the total number of classes is too small to satisfy this condition, an “other” class that is mutually exclusive with all classes should be artificial constructed, or the labels that do not satisfy the condition should be excluded to ensure this prerequisite. The SRR is only suitable for hierarchical label classification because the symbiotic classes are the dependent child nodes of a parent node, which necessarily creates a hierarchical affiliation between labels. Strictly speaking, we only used 0 s and 1 s in the multi-label correlation matrix, which are absolutely mutually exclusive and symbiotic. Values between 0 and 1, we have not yet found suitable loss function to utilize them, and them may be more suitable in neural networks, which could complement our work.

Although our approach exploits the mutually exclusive–symbiotic relationship between multiple hierarchical labels and uses the multi-expert aggregated method to improve generalization performance, there is still room for improvement. The total number of labels that can be recognized by our model is 254. Of the 191,804 samples, more than a quarter of the classes have a sample size of less than 500, and more than half have a sample size of <1400. This makes the contribution of our strategy at the algorithmic level in mitigating data imbalances limited. Combined with the difficulty of the classification task itself, this results in inferior classification performance for some labels. The fundamental solution to this problem is to collect more data, especially for rare classes. This work will continue well into the future. In addition, the detection of feature points in physiological waveforms, such as the position and amplitude of the P wave, QRS complex, and T wave, as well as the traditional quantitative indices based on them, provide very useful domain knowledge, because ECGs are diagnosed on the basis of these quantitative indices. We will build on the already developed U-Net^43^, which uses temporal prior constraints that can successfully detect most physiological waveform locations of wearable ECGs, and fuse traditional quantitative indices into the diagnostic model. This will combine the strengths of both ECG diagnostics and deep learning, resulting in richer representations and better diagnostic performance.

The recently developed ChatGPT^44^ can serve a variety of general-purpose text and image tasks^45^, such as question-and-answer, writing, translating, and designing. Several models serving specialized fields^46^ have also been developed, such as for medical image segmentation, medical information question answering, and legal services. Using a large number of fine-grained annotated ECGs as the basis, we will develop a model based on knowledge of ECG diagnostics that can really understand ECGs, answer questions from both cardiologists and patients, and make appropriate recommendations. Therefore, there is still a lot of work to be done in this field. In the scientific spirit of truthfulness, pragmatism, and innovation, and maintaining close cooperation between medicine and engineering, the performance of intelligent diagnostic models should be continuously improved to meet the needs and expectations of society.

## Methods

### Ethical statement

This study was approved by the Medical Ethics Committee of Chinese PLA General Hospital with the number S2019-318-03, and informed consent was obtained from the participants.

### Local softmax

The most commonly used loss in multi-label classification tasks is the binary cross-entropy (BCE), and for objective comparison, the loss in all of our experiments is the BCE defined as Eq. (1):

1 $${L}_{{BCE}}=-\frac{1}{C}\mathop{\sum}\limits_{i=1}^{C}{w}_{i}\cdot \left[{y}_{i}\cdot \log \left({\sigma }_{i}\right)+\left(1-{y}_{i}\right)\cdot \log \left({1-\sigma }_{i}\right)\right]$$

where $C$ is the total number of classes; ${w}_{i}$ is the pre-set weight of class $i$, which is generally calculated as $N/{N}_{i}$, and $N$ is the number of all samples, ${N}_{i}$ is the number of positive samples of the ${i}_{{th}}$ class; ${y}_{i}$ is the ground truth of the ${i}_{{th}}$ class; and ${\sigma }_{i}$ is the prediction from model mapped to a value range of 0–1 by the activation function.

The baseline of the activation function $\sigma$ for multi-label classification tasks is generally the sigmoid, which is defined as Eq. (2):

2 $${{Sigmoid}}_{i}=\frac{1}{1+{e}^{-{z}_{i}}},$$

where ${z}_{i}$ is the output of model for class $i$. It can be seen that ${{Sigmoid}}_{i}$ is only related to ${z}_{i}$ and is independent of the predicted values from model for the rest of the class.

For multi-class classification tasks, the activation function $\sigma$ is generally the softmax defined as Eq. (3):

3 $${{Softmax}}_{i}=\frac{{e}^{{z}_{i}}}{{\sum }_{j=1}^{C}{e}^{{z}_{j}}}$$

it can be seen that ${{Softmax}}_{i}$ is not only related to ${z}_{i}$, but also to all the remaining $C-1$ classes that are mutually exclusive with class $i$, and ${z}_{i}$ will suppress output values of all classes except it. When softmax is used for multi-label classification, for multiple positive labels in the same sample, the gradients can conflict severely during optimization, resulting in much worse performance than sigmoid.

Concurrent softmax can be used in multi-label object detection tasks defined as Eq. (4):

4 $${{Concurrent\,Softmax}}_{i}=\frac{{e}^{{z}_{i}}}{{\sum }_{j=1}^{C}{(1-{r}_{{ij}})\cdot e}^{{z}_{j}}+{e}^{{z}_{i}}},{with}\,{r}_{{ij}}=\frac{{N}_{{ij}}}{{N}_{j}},$$

where ${r}_{{ij}}$ is the conditional probability that a sample is known to belong to class $j$ and that it also belongs to class $i$, and this value is calculated statistically based on the annotation of the dataset; ${N}_{{ij}}$ is the number of samples both belonging to class $i$ and class $j$, and ${N}_{j}$ is the number of samples in class $j$. When ${r}_{{ij}}=0$, class $i$ is mutually exclusive with class $j$, at which point concurrent softmax degenerates to softmax for these two classes; when ${r}_{{ij}}=1$, the output of the ${j}_{{th}}$ class disappears from the denominator, and the ${i}_{{th}}$ class will not suppress the ${j}_{{th}}$ class. It can be seen that concurrent softmax alleviates the suppression of ${z}_{i}$ on ${z}_{j}$ according to ${r}_{{ij}}$, but this suppression does not disappear completely. When the ground truth value of class $i$ and class $j$ is 1 at the same time, the gradients of the two classes still conflict in the optimization procedure, and it is hard for the model to obtain the optimal solution in the parameter space.

Therefore, we improve the concurrent softmax as Eq. (5):

5 $${{Concurrent\,Softmax}}_{i}^{* }=\frac{{e}^{{z}_{i}}}{{\sum }_{j=1}^{C}{{(1-{r}_{{ij}})}^{\tau }\cdot e}^{{z}_{j}}+{e}^{{z}_{i}}},$$

where $\tau$ is a hyperparameter that we set as its value increases, it causes ${z}_{i}$ to gradually reduce the suppression of the output from these classes related with class $i$.

When taking a particular paradigm $\tau =\infty$, the suppression of ${z}_{i}$ on the classes related to it disappears completely, and only these classes that are completely mutually exclusive with it are suppressed, which we call local softmax as Eq. (6):

6 $${{Local\,Softmax}}_{i}=\frac{{e}^{{z}_{i}}}{{\sum }_{j=1}^{C}{{r}_{{ij}}^{{ME}}\cdot e}^{{z}_{j}}+{e}^{{z}_{i}}},{with}\,{{r}}_{{ij}}^{{ME}}={(1-{r}_{{ij}})}^{\infty }$$

Compared to softmax, the suppression of the remaining classes by class $i$ is reduced from the global $C-1$ classes to the local ${C}_{i}^{{ME}}$ classes, where ${C}_{i}^{{ME}}={\sum }_{j=1}^{C}{r}_{{ij}}^{{ME}}$, which can be exploited the mutually exclusive relation rationally in the loss of multi-label classification tasks. When the model determines that a sample belongs to class $i$, the probability of belonging to its mutually exclusive class $j$ will be suppressed to a very small value, reducing the likelihood that the sample will be predicted to be in both mutually exclusive classes at the same time, and making the output of the model more reasonable.

### Symbiotic ranking regularizer

There are mutually symbiotic relationships in hierarchical labels, in which a parent node governs several child nodes. Our motivation is as follows: when the truth value of a child node is 1, make the predicted value of model for its parent node also 1 according to the symbiotic correlation; when the truth value of the parent node is 0, make the predicted value of model for all its child nodes are 0. We use pairwise ranking loss to implement SRR, which is defined as Eq. (7):

7 $${{Regularizer}}_{{MS}}=\log \left[1+\mathop{\sum}\limits_{i=1}^{C}\mathop{\sum}\limits_{j=1}^{{r}_{i}^{{MS}}}\exp ({\sigma }_{j}-{\sigma }_{i})\right],{with}\,{r}_{{ij}}^{{MS}}={r}_{{ij}}^{\infty }-E\left(C\right),$$

where $i$ is the parent node, $j$ is the child node, and $E$ is the unit matrix. In mathematical calculations, $i$ traverses all the classes. When traversed to a parent node, ${r}_{i}^{{MS}}$ is all its child nodes; when traversed to a child node, ${r}_{i}^{{MS}}$ is an all-0 vector of length $C$.

### Ensemble

In ensemble learning, a strong learner is obtained by training some “good and different” weak base learners, which would make errors on different samples or different classes, and then aggregating these base learners, which can be erudite and reduce the variance of the final output. The core of SADE is to use long-tailed, balanced, and inverse losses to train the model and then aggregate three results, which can mitigate the data imbalance and cope with test sets with various unknown distributions. However, SADE was developed to deal with multi-class classification problems where the loss is softmax, thus we improved it using sigmoid and local softmax to make it suitable for multi-label classification tasks.

When the activation function $\sigma$ is sigmoid, its three functional variants of long-tailed, balanced, and inverse are defined as Eqs. (8–10):

8 $${\sigma }_{i}^{L-{Sigmoid}}={Sigmoid}\left({z}_{i}\right),$$

9 $${\sigma }_{i}^{B-{Sigmoid}}={Sigmoid}\left({z}_{i}-{v}_{i}^{1}\right),{with}\,{{v}}_{i}^{1}=\log \left(\frac{N}{{N}_{i}}-1\right),$$

10 $${\sigma }_{i}^{I-{Sigmoid}}={Sigmoid}\left({z}_{i}-{v}_{i}^{1}+{v}_{i}^{2}\right),{with}\,{{v}}_{i}^{2}={Inversed}\left({v}_{i}^{1}\right),$$

where $N$ is the total number of samples in the dataset and ${N}_{i}$ is the number of samples in class $i$. ${Inversed}$ is a function and transforming the balanced prior ${v}_{i}^{1}$ to the inversed prior ${v}_{i}^{2}$ by sorting it in inverse order according to the ${N}_{i}$, which simulates the head classes of the long-tailed distribution into tail classes, first transforming the long-tailed distribution into the balanced distribution and then into the inversed distribution.

When the activation function $\sigma$ is local softmax, its three functional variants of long-tailed, balanced, and inverse are defined as Eqs. (11–13):

11 $${\sigma }_{i}^{L-{Local\,Softmax}}={Local\,Softmax}\left({z}_{i}\right),$$

12 $${\sigma }_{i}^{B-{Local}\,{Softmax}}={{Local}\,{Softmax}}\left({z}_{i}+{\pi }_{i}^{1}\right),{with}\,{\pi }_{i}^{1}=\log \left(\frac{{N}_{i}}{N}\right),$$

13 $${\sigma }_{i}^{I-{{Local}\,{Softmax}}}={{Local}\,{Softmax}}\left({z}_{i}+{\pi }_{i}^{1}-{\pi }_{i}^{2}\right),{with}\,{\pi }_{i}^{2}={Inversed}\left({\pi }_{i}^{1}\right)$$

similarly, ${\pi }_{i}^{1}$ and ${\pi }_{i}^{2}$ are the balanced prior and inversed prior of the local softmax, respectively.

We use the multi-scale convolutional network of 18 layers with the same architecture but different losses as homogenized base learners, and setting the number of experts to be 12 means that there are a total of 12 losses: for each of the four losses sigmoid, symbiotic sigmoid, local softmax, and symbiotic local softmax, there are three derivations of long-tailed, balanced and inverse logit adjusted ways. The final loss function is the sum of these 12 losses, which is defined as Eq. (14):

14 $$\begin{array}{lll}{L}_{1}\;\,={BCE}\left({y,\sigma }^{L-{Sigmoid}}\left({z}_{1}\right)\right),\\ {L}_{2}\;\,={BCE}\left(y,{\sigma }^{B-{Sigmoid}}\left({z}_{2}\right)\right)\\ {L}_{3}\;\,={BCE}\left(y,{\sigma }^{I-{Sigmoid}}\left({z}_{3}\right)\right)\\ {L}_{4}\;\,={BCE}\left({y,\sigma }^{L-{Sigmoid}}\left({z}_{4}\right)\right)+{{Regularizer}}_{{MS}}\left({z}_{4}\right),\\ {L}_{5}\;\,={BCE}\left({y,\sigma }^{B-{Sigmoid}}\left({z}_{5}\right)\right)+{{Regularizer}}_{{MS}}\left({z}_{5}\right),\\ {L}_{6}\;\,={BCE}\left(y,{\sigma }^{I-{Sigmoid}}\left({z}_{6}\right)\right)+{{Regularizer}}_{{MS}}\left({z}_{6}\right),\\{L}_{7}\;\,={BCE}\left({y,\sigma }^{L-{{Local}\,{Softmax}}}\left({z}_{7}\right)\right),\\ {L}_{8}\;\,={BCE}\left(y,{\sigma }^{B-{{Local}\,{Softmax}}}\left({z}_{8}\right)\right),\\{L}_{9}\;\,={BCE}\left(y,{\sigma }^{I-{{Local}\,{Softmax}}}\left({z}_{9}\right)\right),\\ {L}_{10}={BCE}\left({y,\sigma }^{L-{{Local}\,{Softmax}}}\left({z}_{10}\right)\right)+{{Regularizer}}_{{MS}}\left({z}_{10}\right),\\ {L}_{11}={BCE}\left({y,\sigma }^{B-{{Local}\,{Softmax}}}\left({z}_{11}\right)\right)+{{Regularizer}}_{{MS}}\left({z}_{11}\right),\\ {L}_{12}={BCE}\left(y,{\sigma }^{I-{{Local}\,{Softmax}}}\left({z}_{12}\right)\right)+{{Regularizer}}_{{MS}}\left({z}_{12}\right),\\ L\;\;\,=\mathop{\sum}\limits_{i=1}^{12}{L}_{i},\end{array}$$

where $y$ is the ground truth of the ECG data and ${z}_{1}$–${z}_{12}$ are the predicted values output by different experts. For the model training, we have two options as in Fig. 3: the first one is to train 12 models via 12 losses respectively. The advantage of this scheme is its preferable classification performance, but the operation is tedious and obviously increasing training time, memory, and inference time. The preference for classification performance led us to choose this scheme. The second is to train only a multi-output model whose final loss function is the sum of these 12 losses ${\sum }_{i=1}^{12}{L}_{i}$. The performance of this scheme will be slightly worse than the first one, but the operation is simple, and the amount of computer memory and computation is significantly reduced compared to the former, which is suitable for scenarios where computational resources are limited, and there is a time constraint for inference. For the 12 predictions-aggregated, we fuse them in the way of simple averaging to get the final result.

### Network architecture

Here, two convolutional network architectures, a single-expert network, and a multi-expert network, cope with the two ensemble schemes. All layers and operations in both networks are one-dimensional, and their parameters are 2.07 M and 12.78 M, respectively. The convolution layer is multi-scaled, which consists of four convolution kernels of different lengths 3, 5, 9, 17 are computed in parallel, then their results are concatenated channel-wise to obtain a more diverse receptive field. A standard convolution module is set up as MSConv-BN-RELU, they are Multi-Scale Convolution, Batch Normalization and Rectified Linear Unit. A residual connected block is composed of two convolution modules whose stride is set to 2, and a dropout layer with a rate is 0.2.

The single-expert network is shown in Fig. 3, its input is an ECG with a length of 7500 digital samples and 12 channels, and its output is a 254-dimensional vector. The initial number of channels of the network is 12, which is also the number of leads in the ECG. The channels of the backbone are set to 64 + 16k, where k is the index of the residual block, and there are 8 of them. The index k is starting from 0, and the number of channels increases by 16 for each residual block through which the data flows. Finally, a representation vector of the data is obtained by a global average pooling operation, and this vector is mapped to 254 dimensions by the linear layer, which is the total number of classes to be classified.

The multi-expert network uses the same MSDNN architecture, except that it has multiple classification heads corresponding to 12 expert losses. Its input also is an ECG in the same format, and its output is 12 stacked 254-dimensional vectors. The shared backbone consists of six residual blocks. Each loss has its own independent classification header: two residual blocks and a linear connection layer. The network uses multiple experts to get predictions for 12 views of the same ECG to compute the loss separately, and the total loss in training is the sum of these 12 sub-losses.

Our code and experiments were implemented on pytorch 1.17. The network parameters were initialized randomly by Kaiming initialization. The model has trained 130 epochs, with the optimizer being SGD, momentum set to 0.9, and weight decay to 1e-5. The default learning rate is 0.01, and its schedule uses the combination of one-cycle and three sawtooth annealing warm restarts. The learning rate increases linearly from 0.001 to 0.01 for the first 45 epochs, then decays linearly to 0.001 for the next 45 epochs, and rapidly decays to 1e-6 for the next 10 epochs. For the last 30 epochs, we set up 3 sawtooths, each lasting for 10 epochs at the start, the learning rate was 0.005 and then decayed rapidly to 1e-6, the purpose of which is to try to make the model jump out of the local optimal solution in the parameter space, and restart the convergence process a few more times in order to get the globally optimal solution. We save the model with the best average AUPRC performance on the test set during training.

### Statistical analysis

We performed paired samples *t* tests on the following sets of models using AUPRC as the indicator to analyze the effectiveness of our methodology, and statistically significant differences between models exist when the *p* value is less than the significance level $\alpha$ of 0.05. The *p* values of Softmax vs Local Softmax and Sigmoid vs Local Softmax are 1.32 × 10^−19^ and 0.859, respectively, which shown that our local softmax outperforms the vanilla softmax and reaches a level comparable to the sigmoid in multi-label classification tasks. The *p* values of Sigmoid vs Symbiotic Sigmoid and Local Softmax vs Symbiotic Local Softmax are 0.123 and 0.632, respectively, both larger than $\alpha$, indicating that the SRR has little contribution to improving the classification performance when it is used alone. However, the *p* values of Sigmoid vs Sigmoid+Local Softmax and Sigmoid vs Sigmoid+Symbiotic Sigmoid are 4.15 × 10^−16^ and 5.05 × 10^−20^ respectively both much smaller than $\alpha$, which shown that local softmax and SRR have different concerns from sigmoid, they are truly “good but different”, and their combination can actually improve the classification performance of the model. Finally, there is a statistical comparison between before and after applying MESEL on the 6 approaches in Table 3, the *p* values are 5.03 × 10^−13^, 2.03 × 10^−14^, 2.62 × 10^−14^, 4.63 × 10^−11^, 4.50 × 10^-7^, and 5.11 × 10^−10^ respectively, all of them smaller than $\alpha$, indicating that our MESEL is not only compatible and can be adapted to existing deep learning approaches, but also can continue to improve the diagnostic performance of intelligent models on top of the existing ones.

## Supplementary information

Supplementary Information

## Data Availability

The ECG recordings and models used in this paper cannot be publicly available due to their commercial nature. Source data and Supplementary Table are provided with this paper.

## Code availability

The source code is available at GitHub: https://github.com/SMU-MedicalVision/MESEL.
